# Commissioning and validation of BrainLAB cones for 6X FFF and 10X FFF beams on a Varian TrueBeam STx

**DOI:** 10.1120/jacmp.v14i6.4493

**Published:** 2013-11-04

**Authors:** David B. Wiant, Jonathon A. Terrell, Jacqueline M. Maurer, Caroline L. Yount, Benjamin J. Sintay

**Affiliations:** ^1^ Department of Radiation Oncology Cone Health Cancer Center Greensboro NC USA

**Keywords:** flattening filter free, stereotactic radiosurgery, circular collimator, cones

## Abstract

Small field dosimetry is a challenging task. The difficulties of small field measurements, particularly stereotactic field size measurements, are highlighted by the large interinstitution variability that can be observed for circular cone collimator commissioning measurements. We believe the best way to improve the consistency of small field measurements is to clearly document and share the results of small field measurements. In this work we report on the commissioning and validation of a BrainLAB cone system for 6 MV and 10 MV flattening filter‐free (FFF) beams on a Varian TrueBeam STx. Commissioning measurements consisted of output factors, percent depth dose, and off‐axis factor measurements with a diode. Validation measurements were made in a polystyrene slab phantom at depths of 5 cm, 10 cm, and 15 cm using radiochromic film. Output factors for the 6xFFF cones are 0.689, 0. 790, 0.830, 0.871, 0.890, and 0.901 for 4 mm, 6 mm, 7.5 mm, 10 mm, 12.5 mm, and the 15 mm cones, respectively. Output factors for the 10xFFF cones are 0.566, 0. 699, 0.756, 0.826, 0.864, and 0.888 for 4 mm, 6 mm, 7.5 mm, 10 mm, 12.5 mm, and the 15 mm cones, respectively. The full width half maximum values of the off‐axis factors agreed with the nominal cone size to within 0.5 mm. Validation measurements showed an agreement of absolute dose between calculation and plan of ≤ 3.6%, and an agreement of field sizes of ≤ 0.3 mm in all cases. Radiochromic film validation measurements show reasonable agreement with beam models for circular collimators based on diode commissioning measurements.

PACS numbers: 87.53.Ly, 87.53.Bn, 87.56.nk, 87.55.D‐, 87.55.km

## I. INTRODUCTION

Recently, flattening filter‐free (FFF) treatment beams have become commercially available on several platforms, including the Varian TrueBeam (Varian Medical Systems, Palo Alto, CA). Flattening filter‐free beams may offer a number of advantages compared to conventional flattened beams, such as increased dose rate, decreased out of field scatter dose, and decreased neutron production.^(1)^ The increased dose rate of the FFF beams is the attribute that has garnered the most clinical attention. The high dose rate of the FFF beams may have the largest impact on stereotactic radiosurgery (SRS), stereotactic body radiotherapy (SBRT), and intensity‐modulated radiation therapy (IMRT). These treatments generally utilize restrictive immobilization devices to limit patient motion and maximize setup reproducibility. The high‐dose rate FFF beams may lead very directly to a reduction in treatment time, which could limit patient motion and decrease patient discomfort.^(^ ^2^ ^,^ ^3^ ^)^ The use of FFF beams may be most beneficial in circular collimator‐ or cone‐based SRS treatments that usually employ uncomfortable head frames or masks and have high numbers of monitor units per field (due to the low output factors for cones).

A good deal of work has been done on the modeling, measurement, and characterization of FFF beams in general^4^, ^5^, ^6^, ^7^, ^8^, ^9^, ^10^, ^11^, ^12^, ^13^, ^14^ and in characterization of FFF beams on the TrueBeam.^15^, ^16^, ^17^, ^18^ However, there is a paucity of published data on the characterization of SRS cones,^19^, ^20^, ^21^, ^22^ and of SRS cones coupled with FFF beams in particular. Small field commissioning and verification measurements are technically challenging^(^ ^23^ ^,^ ^24^ ^)^ and have been shown to vary among institutions.^(25)^

In that light, we present commissioning and validation measurements for 4 mm, 6 mm, 7.5 mm, 10 mm, 12.5 mm, and 15 mm diameter BrainLAB SRS cones (BrainLAB AG, Feldkirchen, Germany) for the BrainLAB iPlan treatment planning system on a TrueBeam STx for 6xFFF and 10xFFF energies.

## II. MATERIALS AND METHODS

### A. Commissioning

Data were acquired for the commissioning of the iPlan 4.5 treatment planning system according to vendor‐specific recommendations.^(26)^ The iPlan circular cone planning system uses a tissue‐maximum ratio algorithm based on measured percent depth‐dose (PDD) curves, off‐axis ratios (OAR), and scatter factors ($S_{t}$). The circular cone algorithm accounts for heterogeneities in the depth dimension by means of radiological depths determined from the CT scan. The algorithm assumes secondary scatter is of limited importance and does not explicitly account for it in the dose calculation. The machine was calibrated with a source‐to‐surface distance (SSD) setup using a 0.6 cc volume Farmer‐type chamber following TG‐51 guidelines.^(27)^ For each beam energy, one monitor unit was set to deliver 1 cGy to the depth of maximum dose ($d_{\text{max}}$) in water at 100 SSD for a 10cm×10cm field. The output for a 10cm×10cm field at a depth of 10 cm in water at 100 cm SSD was then determined for each energy from PDD curves that were measured with a PTW Semiflex 0.125 cc volume chamber (PTW GmBH, Friedberg, Germany) in a PTW MP3 scanning water phantom.

For each cone size the OAR, the PDD and the St were measured using a Sun Nuclear Edge Detector diode (Sun Nuclear Corp., Melboune, FL) in a PTW MP3 phantom. The Edge Detector diode has an active volume of $0.8\;\text{mm}\times 0.8\;\text{mm}\times 0.03\;\text{mm}\;(0.0019\;{\text{mm}}^{3})$. The diode is housed in a 0.13 mm rectangular brass casing with an epoxy fill. The jaws were set to form a 2cm×2cm square field for all cone measurements. The phantom was leveled and it was verified that the diode travel was parallel and perpendicular to the water surface. The OAR measurements were made at 92.5 cm SSD at a depth of 7.5 cm. The surface of the diode was leveled to the water surface then shifted 0.3 mm above the water surface so that the effective point of measurement was at the water surface; this was set as zero depth. The diode was moved to a depth of 7.5 cm. For each cone, in‐plane and cross‐plane profiles were initially acquired that were used to locate the diode at the radiological center of the cone at the scanning depth. With the diode set at the radiological center of the cone, cross‐plane and in‐plane profiles were acquired with a 0.5 mm step size covering ±30mm in each plane. The reported half profiles used for commissioning are averages of these measurements.

The PDD measurements were made at $100\;\text{cm}-d_{\text{max}}$ SSD for each cone and energy. The diode was offset 0.3 mm from the surface, as described above. Before each cone measurement, cross‐plane and in‐plane profiles were acquired at depths of 10 cm and 20 cm to verify that the diode was at the radiological center of the cone and that the travel in the depth dimension was perpendicular to the water surface. Measurements were made from a depth of 300 mm up to 50 mm in 5 mm steps, then from 50 mm to 0 mm in 1 mm steps.

The output factors were measured at $d_{\text{max}}$ for each energy at a SSD of $100\;\text{cm}-d_{\text{max}}$. Again, the diode was offset 0.3 mm from the surface. Before each cone measurement, cross‐plane and in‐plane profiles were acquired to verify the diode was at the radiological center of the cone. Note the cone output measurements were referenced directly to a measurement with the diode for a 10cm×10cm field at 100 cm SSD at a depth of 10 cm. The so called “daisy chaining” method (which involves the comparison of diode and ion chamber measurements at a field size intermediate between the stereotactic field sizes and the 10 cm field commonly used for machine calibration^(20)^) was not used as ratios of a 5cm×5cm field to a 10cm×10cm field in the output factor setup gave comparable results for diode and Farmer chamber measurements. This is in agreement with previously reported findings.^(20)^

The flattened 6x cones were commissioned in the same manner as described above. For comparison sake, the results of the 6x measurements will be presented as well.

### B. Validation

Validation measurements were made with GAFCHROMIC EBT3 film (Ashland Inc., Covington, KY) in a polystyrene slab phantom and a Lucy 3D QA Phantom (Standard Imaging Inc., Middleton, WI) with film insert. The slab phantom consisted of four pieces of polystyrene stacked on top of each other with film between the slabs at depths of 5 cm, 10 cm, and 15 cm. The phantom was scanned on a Philips Brilliance Big Bore CT scanner (Philips Healthcare, Andover, MA) with 1 mm slice thickness. Treatment plans were created in iPlan for each cone and energy combination. For each plan the isocenter was set at the center of the middle (10 cm depth) film. A single static beam with a gantry and collimator angles of zero was planned to give a dose of 7 Gy to isocenter. The CT and dose were exported in DICOM format to a custom MATLAB (The MathWorks Inc., Natick, MA) program for analysis.

**Figure 1:**
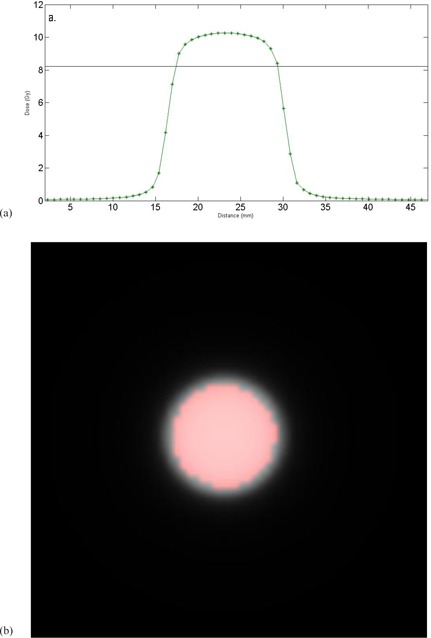
(a) A 1D example of how the dose was determined. A profile through the center of a calculated plan dose is shown. The solid line shows 80% of the maximum dose. All pixels above the line would be used to determine the absolute dose to use for comparison. (b) A representative calculated dose plane. The red circle shows the pixels with ≥80% of the maximum dose in the plane.

In the software, the dose and CT were reoriented to show slices through the film planes. The dose was exported with voxel size equal to the CT voxel size (0.772mm×0.772mm×1.mm). In the software, the dose voxels were interpolated up to the size of the film pixels (0.169mm×0.169mm×0.169mm). The centroid of the dose area was determined for the calculated dose in each film plane and for the measured doses on the corresponding films. The calculated doses and the measured film doses were registered based on centroid position, which implicitly assumed rotational symmetry of both dose distributions. Each registered calculated‐measured dose pair was compared by full width half maximums (FWHM) of profiles through the center of the dose pair and by absolute dose. The absolute dose was evaluated by taking the average of the voxels on the calculated dose that had values >80% of the maximum calculated dose and comparing those to the average of the corresponding voxels on the registered measured film dose (Fig. 1).

A cutoff of 80% of the maximum dose was chosen to include the high‐dose region and exclude the penumbra region, often defined as 20%‐80% isodose lines for SRS.^(28)^ An average was used instead of the more commonly used gamma analysis^(29)^ because analysis with a small distance to agreement would provide too “loose” a tolerance for stereotactic field sizes (e.g., a 1 mm distance to agreement would look across a quarter of the 4 mm field), and any absolute dose criteria with 0 mm distance to agreement would provide too tight a tolerance (it would not allow for any misalignment of the measured ‐ calculated dose registration or any noise in the film response).

The EBT3 film used for these measurements was purchased in 35.56cm×43.18cm sheets (lot A09231103). The film was cut into 4cmx4cm squares >24hours before irradiation. The upper left corner was marked at the time the film was cut to define orientation. The film was calibrated in a slab polystyrene phantom that consisted of two 5 cm thick pieces with a chamber hole for an Exradin A1SL (Standard Imaging) drilled 5 mm deeper than the film plane. The phantom was scanned on a Philips Big Bore scanner. Plans were created in Varian Eclipse 10.0 with the isocenter set to the film plane that delivered 50, 100, 200, 300, 400, 600, 800, 1000, and 1200 cGy to the film with a 6x beam. The doses to the chamber were also recorded and used to verify machine output at the time of film calibration. The phantom was set up on the TrueBeam STx with the film plane at isocenter. Identical film orientation was used for each field. Chamber dose was recorded for each film.

**Figure 2:**
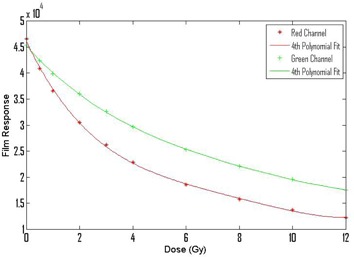
The stars and pluses show responses of the calibration films as a function of dose for the red and green channels. The solid lines show fits of 4th degree polynomials to the calibration data.

The film was stored in a dark, dry environment for24±1 hour after irradiation. The film was then scanned with a resolution of 150 dpi and 48‐bit depth at the center of an Epson Expression 10000 XL flatbed scanner (Epson America Inc., Long Beach, CA) with each piece of film in the same orientation. The film images were stored as uncompressed TIFF files. The film files were analyzed in custom MATLAB software. A 0.5cm×0.5cm region of interest was placed at the center of the film. The average response of the red and green channels in this region was recorded for each piece of irradiated film and for an unirradiated film. The film response was plotted as a function of dose for the red and green channels. Both the red and green color channels were used as the red channel shows greater sensitivity at low doses, while the green channel has a larger dynamic range than the red channel.^(30)^ The dose response curves were fit with fourth order polynomials (Fig. 2). The fourth order polynomials were used to convert film response to dose for all film measurements. Note, the chamber measurements agreed with calculated measurements to <1% for all film calibration irradiations.

For the validation measurements, EBT3 was placed at depths of 5 cm, 10 cm, and 15 cm in the slab phantom described above. The phantom was set up on the TrueBeam STx and positioned by cone‐beam CT. The phantom was then irradiated for all combinations of cone size and energy two separate times. These films were processed in the same manner as the calibration films. The films were scanned and sent to the MATLAB software where profiles and absolute dose were determined for the red and green channels, as described above. No smoothing was applied to the films in the MATLAB analysis. All results shown are the average of the two film measurements.

The Lucy phantom was scanned on a Philips Brilliance Big Bore CT scanner with 1 mm slice thickness with the film plane oriented parallel to the CT couch. Treatment plans were created in iPlan for each cone and energy combination with the isocenter set at the center of the film plane. A single arc traveling counterclockwise from 175° to 185° was planned to deliver about 7.5 Gy to isocenter for each energy and cone combination. The Lucy phantom was mounted on a base using the neck extension so that the actual phantom could be hanged over the end of the treatment couch, so a couch was not included in the dose calculation. After the plans were completed, the CT and dose were exported in DICOM format to a custom MATLAB program for analysis.

The Lucy phantom was set up on the TrueBeam STx in the same configuration that was used when the CT was acquired. A cone‐beam CT was then acquired to position the phantom on the treatment machine. Each of the plans was delivered two times.

The film handling and analysis were similar to what was described above. The two differences to the process were: 1) the film was registered to the planned dose in the MATLAB software using fiducial marks that were left in the corners of the film by the Lucy phantom, and 2) profiles were only acquired in the direction perpendicular to the direction of gantry travel. Absolute dose for the red and green color channels was calculated using voxels ≥80% of the maximum planned dose, as described above. Absolute dose and profile measurements are reported for each of the arc measurements.

Immediately following the irradiations of each of the validation phantom, the film calibration phantom (described above) was set up with film and ion chamber. A 10cm×10cm field was delivered using the 6xFFF and 10xFFF beams. The film was processed as described above. The ion chamber measurements were used to verify machine output at the time of the validation measurements, while the film measurements were used to verify film response for the particular sheet of film used in the validation measurements.

## III. RESULTS

### A. Commissioning

**Figure 3:**
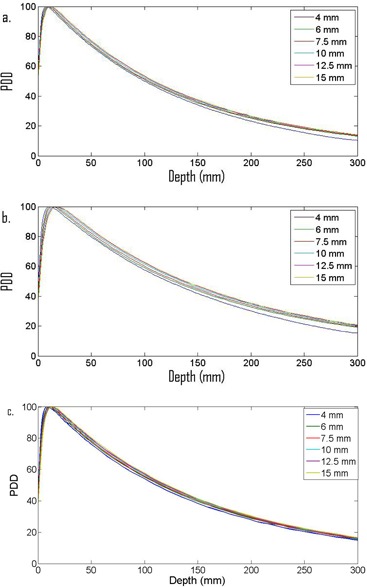
PDD data for (a) 6xFFF and (b) 10xFFF cones, and for (c) flattened 6x.

The output for a 10cm×10cm field at a depth of 10 cm in water at 100 cm SSD was 0.631 cGy/monitor unit for the 6xFFF beam and 0.708 cGy/monitor unit for the 10xFFF beam. The PDD measurements are shown in Fig. 3. The $d_{\text{max}}$ for the 6xFFF beam for the reference setup is 13 mm, $d_{\text{max}}$ for the 10xFFF beam for the reference setup is 21 mm. Measured $d_{\text{max}}$ for the 6xFFF cones ranges from 8 mm for the 4 mm cone to 12 mm for the 15 mm cone. Measured $d_{\text{max}}$ for the 10xFFF cones ranges from 11 mm for the 4 mm cone to 18 mm for the 15 mm cone.

The flattened 6x cones had an output of 0.663 cGy/monitor unit in the calibration setup. The $d_{\text{max}}$ for the 6x beam in the reference setup is 14 mm. Measured $d_{\text{max}}$ for the 6x cones ranges from 8 mm for the 4 mm cone to 14 mm for the 15 mm cone.

**Figure 4:**
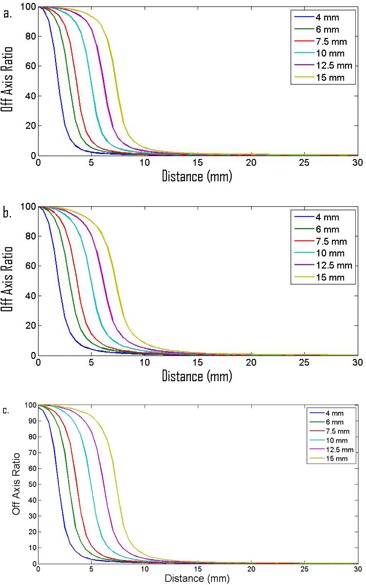
PDD data for (a) 6xFFF and (b) 10xFFF cones, and for (c) flattened 6x.

The OARs are shown in Fig. 4. The FHWM for the 6xFFF cones are 3.8 mm, 5.8 mm, 7.2 mm, 9.9 mm, 12.3 mm, 14.6 mm, and for the 4 mm, 6 mm, 7.5 mm, 10 mm, 12.5 mm, and the 15 mm cones, respectively. The FHWM for the 10xFFF cones are 3.8 mm, 5.9 mm, 7.3 mm, 9.9 mm, 12.2 mm, and 14.5 mm for the 4 mm, 6 mm, 7.5 mm, 10 mm, 12.5 mm, and the 15 mm cones, respectively. The FHWM for the 6x cones are 3.8 mm, 5.8 mm, 7.2 mm, 9.9 mm, 12.2 mm, and 14.6 mm for the 4 mm, 6 mm, 7.5 mm, 10 mm, 12.5 mm, and the 15 mm cones, respectively.

The output factors are shown in Table 1. The reported values are the ratio of the cone measurements to a 10cm×10cm field in the output reference setup described above. The output factors for the 6xFFF cones are 0.689, 0.790, 0.830, 0.871, 0.890, and 0.901 for the 4 mm, 6mm, 7.5 mm, 10 mm, 12.5 mm, and the 15 mm cones, respectively. The output factors for the 10xFFF cones are 0.566, 0.699, 0.756, 0.826, 0.864, and 0.888 for the 4 mm, 6 mm, 7.5 mm, 10 mm, 12.5 mm, and the 15 mm cones, respectively. The output factors for the 6x cones are 0. 631, 0.746, 0.795, 0.843, 0.866, and 0.879 for the 4 mm, 6 mm, 7.5 mm, 10 mm, 12.5 mm, and the 15 mm cones, respectively.

**Table 1:** Summary of output factors and FWHM factors measured at commissioning

		*4 mm*	*6 mm*	*7.5 mm*	*10 mm*	*12.5 mm*	*15 mm*
	OUTPUT	0.689	0.790	0.830	0.871	0.890	0.901
	OAR FWHM (mm)	3.8	5.8	7.2	9.9	12.3	14.6
	OUTPUT	0.566	0.699	0.756	0.826	0.864	0.888
	OAR FWHM (mm)	3.8	5.9	7.3	9.9	12.2	14.5
	OUTPUT	0.631	0.746	0.795	0.843	0.866	0.879
	OAR FWHM (mm)	3.8	5.8	7.2	9.9	12.3	14.6

### B. Validation

**Figure 5:**
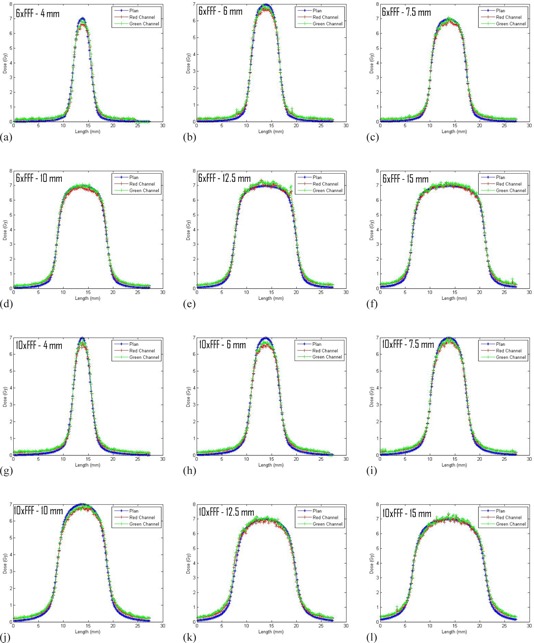
Representative profiles of the static field validation measurements. The figures all show profiles through the film plane at a depth of 10 cm in the horizontal direction.

For each cone and energy, film measurements at depths of 5 cm, 10 cm, and 15 cm were analyzed for the static field validation measurements. Vertical and horizontal profiles, along with absolute doses, were acquired for each calculated dose–measured dose pair for both the red and green color channels (Fig. 5). The FWHM for the profiles and absolute doses are shown in Table 2. The measured FWHM agreed with the calculated FHWM to ≤ 0.3 mm in all cases. The average of the red and green channel absolute doses agreed with the calculated dose to <3.6% in all cases. Note the ion chamber measurements acquired in the film calibration phantom following the verification irradiations showed that the machine output was within 0.5% of the expected values for both the 6xFFF and 10xFFF beams. The films that were irradiated with the ion chamber measurements had responses within 0.5% of the expected values for the 6xFFF and 10xFFF beams. No corrections were made as a result of these checks.

**Figure 6:**
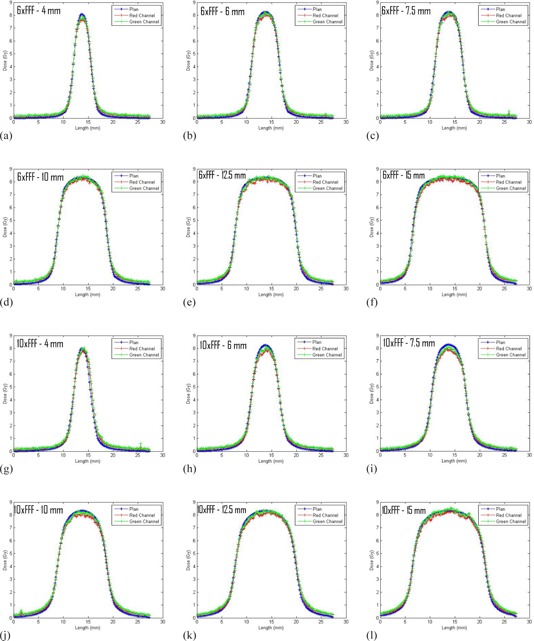
Profiles of the arc validation measurements in the direction perpendicular to gantry travel.

For the arc validation measurements profiles (Fig. 6) and absolute dose comparisons (Table 3) between the planned and measured doses were generated for the red and green color channels The average of the red and green channel absolute doses measurements for the two irradiations show and agreement of −3.9% to −0.3% with the planned doses. Note, measurements made in the calibration setup gave agreement ≤ 0.5% with the expected results for both the 6xFFF and 10xFFF beams.

**Table 2:** Summary of absolute dose and red channel horizontal profile FWHMs data for the validation measurements

		*4 mm*	*6 mm*	*7.5 mm*	*10 mm*	72.5 mm	75 mm
	Dose Plan (Gy)	9.5	9.4	9.4	9.5	9.5	9.5
6XFFF‐5 cm	Dose Red/Green (Gy)	9.4/9.6	9.2/9.3	9.2/9.3	9.4/9.5	9.5/9.6	9.5/9.6
	FWHM Plan (mm)	3.5	5.5	6.8	9.3	11.6	13.8
	FWHM Red (mm)	3.6	5.6	6.9	9.4	11.7	13.9
	Dose Plan (Gy)	6.4	6.4	6.4	6.4	6.5	6.5
6XFFF‐10 cm	Dose Red/Green (Gy)	6.2/6.4	6.2/6.3	6.3/6.4	6.4/6.5	6.5/6.6	6.5/6.6
	FWHM Plan (mm)	3.7	5.8	7.1	9.9	12.3	14.6
	FWHM Red (mm)	3.9	6.0	7.3	10.0	12.3	14.7
	Dose Plan (Gy)	4.2	4.3	4.4	4.4	4.4	4.5
6XFFF‐15 cm	Dose Red/Green (Gy)	4.2/4.3	4.2/4.3	4.2/4.3	4.3/4.4	4.4/4.5	4.4/4.5
	FWHM Plan (mm)	3.9	6.1	7.5	10.4	12.9	15.3
	FWHM Red (mm)	4.1	6.3	7.7	10.5	13.1	15.5
10XFFF‐5 cm	Dose Plan (Gy)	8.8	8.8	8.8	8.8	8.7	8.8
	Dose Red/Green (Gy)	8.7/8.9	8.4/8.6	8.4/8.6	8.5/8.7	8.5/8.7	8.6/88
	FWHM Plan (mm)	3.7	5.6	6.9	9.4	11.6	13.8
	FWHM Red (mm)	3.7	5.6	7.0	9.4	11.7	13.9
10XFFF‐10 cm	Dose Plan (Gy)	6.3	6.4	6.4	6.4	6.4	6.4
	Dose Red/Green (Gy)	6.1/6.3	6.1/6.2	6.2/6.3	6.2/6.3	6.3/6.4	6.4/6.6
	FWHM Plan (mm)	3.8	5.9	7.2	9.9	12.3	14.6
	FWHM Red (mm)	3.9	6.0	7.4	10.0	12.3	14.8
10XFFF‐15 cm	Dose Plan (Gy)	4.5	4.6	4.7	4.7	4.. 7	4.7
	Dose Red/Green (Gy)	4.5/4.6	4.5/4.6	4.6/4.7	4.6/4.7	4.7/4.8	4.7/4.8
	FWHM Plan (mm)	4.0	6.2	7.6	10.4	12.9	15.2
	FWHM Red (mm)	4.2	6.3	7.8	10.5	12.7	15.4

**Table 3:** Summary of absolute dose data for the arc validation measurements

		*4 mm*	*6 mm*	*7.5 mm*	*10 mm*	*12.5 mm*	*15 mm*
	Dose Plan (Gy)	7.4	7.6	7.7	7.8	7.8	7.8
6XFFF	Dose Red (Gy)	7.1	7.4	7.4	7.6	7.7	7.7
	Dose Green (Gy)	7.2	7.5	7.5	7.8	7.8	7.9
	Dose Plan (Gy)	7.3	7.6	7.6	7.7	7.7	7.7
10XFFF	Dose Red (Gy)	7.2	7.2	7.2	7.4	7.5	7.6
	Dose Green (Gy)	7.3	7.4	7.4	7.6	7.6	7.8

## IV. DISCUSSION

**Figure 7:**
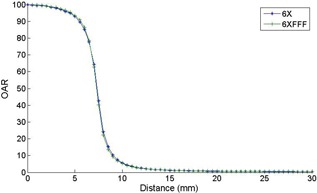
A comparison of the 6xFFF and the flattened 6x OAR commissioning measurements for the 15 mm cone. Negligible difference can be observed between the shapes of the 6x and 6xFFF OARs over the cone sizes measured in this work.

The OARs measured at commissioning are in good agreement with the nominal cone size and they are consistent between the 6xFFF and 10xFFF energies. Also of note is that the 6xFFF OARs are almost identical to the flattened 6x OAR (i.e., the effect of the flattening filter is not evident for these small field sizes (see Fig. 7)). The measured PDDs increase with increasing cone size and $d_{\text{max}}$ increases with increasing cone size for both energies as would be expected for radiosurgical beams.^(31)^ The surface dose for the 6xFFF beams is larger than the surface dose for the 10xFFF beams.

There are no FFF output factors for comparison in the literature. The measured output factors for the 6x with flattening filter cones are within 3% of reported output values measured on a BrainLAB Novalis.^(^ ^19^ ^,^ ^20^ ^,^ ^22^ ^)^ This suggests that the technique used to make the output measurements provided acceptable accuracy. Several reports exist that suggest that diodes may need “correction factors” for volume averaging and water nonequivalence to accurately predict output factors for small fields.^(^ ^19^ ^,^ ^21^ ^)^ Any diode corrections were beyond the scope of this work and were not considered. This work only reports the raw diode measurements used for commissioning.

The output values decrease with increasing energy. The 6xFFF beam should be slightly “softer” than the 6x beam due to the lack of beam hardening. The 6xFFF beam shows the largest output factors at each cone size, followed by the 6x and then the 10xFFF. This finding is in agreement with expectations as electron range increases with increasing energy, which will lead to an increase in “out‐scatter” relative to “in‐scatter” at the point of measurement with increasing energy and decreasing field size. This is observed directly as a reduction of charged particle equilibrium such that the penumbra region begins to have a larger effect across the center of the field.

Thirty‐five of the 36 measured profiles in the slab phantom (three depths for each of the six cone sizes for two energies) agreed within 0.2 mm. The other profile agreed within 0.3 mm. For a scanning resolution of 150 dpi (~0.169mm/pixel), 0.2 mm offsets are on the order of one pixel, the largest 0.3 mm offset is on the order two pixels. This suggests that the profiles measured with the diodes, the film profiles, and the planning system predictions are in very good agreement.

The agreement of the absolute dose measured in the slab phantom on the film with dose calculated by the planning system ranged from −3.6%to2.4%. Only ten of the 36 measured absolute film doses were greater than the calculated doses. An agreement of 3.6% is not unreasonable for difficult, stereotactic field size measurements. However, a systematic low output was evident in the measured film doses, particularly in the 10xFFF measurements where 14 of the 16 measured doses were less than the calculate doses. Also, the agreement between planned and measured dose was poorer for the small cone sizes. For the 6xFFF energy, the three smallest cones had an average agreement between planned and measured of −0.9%, while the three largest cones had an average agreement of 0.2%. For the 10xFFF energy, the three smallest cones had an average agreement of −1.6%, while the largest cones had an average agreement of −0.9%.

Two possible factors contributing to the consistent low dose readings are: 1) an overresponse of the diodes at small field sizes relative to Monte Carlo^(19)^ and fiber optic dosimeter^(21)^ measurements, and 2) an energy dependence of the EBT film leading to an underresponse of film relative to the diodes.^(22)^ (N.B.: the work by Larraga‐Butierrez et al.^(22)^ was done with EBT2 film, but the active layers of EBT2 and EBT3 share a similar composition.^(30)^) Both of these factors would show an increasing effect at smaller cone sizes and higher energy where the finite size of the diode becomes more relevant and any quasi‐electronic equilibrium conditions start to break down, which is in agreement with the results observed in the validation measurements. In light of these known uncertainties associated with small field film dosimetry, a <3.6% variation between measurement and calculation was deemed reasonable.

The arc measurements in the Lucy phantom showed similar trends to those observed in the slab phantom. The profiles were in very good agreement between measured and planned dose and the measured dose was generally lower than the planned dose. The difference between measured and planned dose tended to increase for smaller cone sizes and higher energies. The factors described above that might contribute to the disagreement between planned and measured dose for the static field slab measurements are also applicable to the arc measurements. In addition, setup accuracy may have an impact on the arc measurements. There is a strong dose gradient in the vertical dimension (e.g., a 0.77 mm (one slice) displacement in the vertical dimension of the 4 mm cone 6XFFF planned dose changes the expected dose by ~6%), so a slight misalignment of the phantom could have a large impact on the planned to measured dose agreement. Given the difficulty of the small field measurements, <4% agreement for an end‐to‐end test of the cone system is a very reasonable result.

## V. CONCLUSIONS

Commissioning measurements for the BrainLAB iPlan treatment planning system with circular cone collimators and FFF beams can be adequately carried out with a Sun Nuclear Edge Detector diode. Our results show a high level of consistency within our data and compared well with published data. Validation measurements with EBT3 film show excellent geometric agreement and reasonable dosimetric agreement with the iPlan treatment planning system.
